# Ventral Spinal Cord Displacement After Single-Level Anterior Cervical Corpectomy: A Case Report

**DOI:** 10.7759/cureus.107566

**Published:** 2026-04-23

**Authors:** Bharat R Dave, Saurabh S Kulkarni, Ajay Krishnan, Shivanand C Mayi, Ravi Ranjan Rai, Mirant B Dave, Mikeson Panthackel, Arjit Vashishtha, Amrithesh Singh, Yogenkumar Adodariya

**Affiliations:** 1 Spine Surgery, Stavya Spine Hospital and Research Institute, Ahmedabad, IND; 2 Spine, Bhavnagar Institute of Medical Sciences (BIMS), Bhavnagar, IND; 3 Spine, Stavya Spine Hospital and Research Institute, Ahmedabad, IND

**Keywords:** cervical, corpectomy, postoperative complication, spinal cord herniation, ventral

## Abstract

Spinal cord herniation is a rare condition characterized by protrusion of the spinal cord through a dural defect, typically associated with progressive neurological deficits. However, postoperative alterations at the ventral dural-cord interface may present as cord displacement without a demonstrable dural breach. We report the case of a 44-year-old woman who presented with progressive gait imbalance, neck pain, and right upper limb radiculopathy of four weeks’ duration. Imaging suggested compressive cervical pathology, and the patient underwent anterior cervical corpectomy and stabilization. Postoperatively, the diagnosis of spinal tuberculosis was established based on GeneXpert (GeneXpert® System; Cepheid, Sunnyvale, CA, USA) testing and histopathological examination. Magnetic resonance imaging (MRI) demonstrated ventral displacement of the cervical spinal cord at the operated level without evidence of a definite dural defect. On postoperative day 2, the patient developed mild, new-onset right-hand grip weakness (Medical Research Council (MRC) grade 4/5 in the C7-T1 distribution), which remained non-progressive. In the absence of radiological evidence of true cord herniation and given the stable clinical course, conservative management was adopted along with initiation of anti-tubercular therapy. Serial follow-up imaging demonstrated progressive reappearance of ventral cerebrospinal fluid (CSF) signal anterior to the spinal cord, with resolution of ventral dural adhesions and associated inflammatory tissue. This radiological improvement correlated with clinical stabilization following anti-tubercular therapy and mechanical stabilization of the segment. This case highlights a potential intermediate entity in the spectrum of ventral spinal cord pathology in the setting of spinal tuberculosis and underscores the importance of distinguishing reversible cord displacement from true herniation to avoid unnecessary surgical intervention.

## Introduction

Spinal cord herniation is a rare but well-documented phenomenon. Known etiologies include idiopathic, iatrogenic, and post-traumatic causes [1]. Spinal cord herniation, by definition, occurs due to herniation of the neural contents through a demonstrable dural defect [2]. The existing literature describes a progressively deteriorating clinical course, with most patients presenting with marked neurological deficits or advancing myelopathy necessitating surgical management with dural repair. At one extreme in the spectrum of ventral dural-cord interface anomalies lies true spinal cord herniation, characterized by protrusion of the cord through a dural defect with progressive neurological deterioration. However, less severe alterations may occur when chronic adhesions, postoperative scarring, or focal thinning of the ventral dura exert a tethering effect on the spinal cord without a demonstrable dural breach. In such cases, the cord may appear displaced anteriorly on imaging while remaining contained within the dural sac, often producing a milder or non-progressive clinical course. Thus, ventral displacement of the cord may reflect a relatively benign or early stage in the disease spectrum rather than a definitive cord herniation. Here, we intend to demonstrate a case with suspected ventral cord displacement without a dural defect, which was managed conservatively.

## Case presentation

A 44-year-old woman presented in a wheelchair to the spine outpatient department with imbalance, neck pain radiating to the right upper limb for four weeks, which increased on flexion and extension. The patient had normal ambulation one month before presentation. Over the following weeks, an insidious onset of gait imbalance developed, which gradually progressed; however, at presentation, the patient remained independently ambulatory and could perform activities of daily living. The symptoms were insidious in onset, gradually progressive over one month. Loss of dexterity was noted with slight difficulty in unbuttoning the shirt. A history of fever had been present for two days. New-onset bladder hesitancy had been present for one week. There was no history of small joint involvement or morning stiffness. On neurological examination, upper and lower limb hyperreflexia were brisk, hypertonia was noted, and power was found to be normal. Hand signs such as Hoffmann sign and inverted supinator jerk were positive bilaterally (Table 1). Sensory examination was normal. Bowel/bladder control was intact. Tandem gait test and Romberg test were positive with a NURICK grade 3, and the modified Japanese Orthopedic Association (mJOA) score was 16 at the time of presentation

**Table 1:** Muscle strength and deep tendon reflexes across right and left upper and lower limbs.

Reflexes and strength	Right	Left
Plantar reflex	Upgoing	Upgoing
Knee jerk	Brisk	Brisk
Ankle jerk	Brisk	Brisk
Biceps reflex	3+	3+
Triceps reflex	3+	3+
Bulk	Normal	Normal
Tone		
Upper limb	Hypertonia+	Hypertonia+
Lower limb	Hypertonia +	Hypertonia +
Power		
Shoulder abduction/abduction	5/5	5/5
Elbow-flexion/extension	5/5 5/5	5/5 5/5
Wrist-flexion/extension	5/5 5/5	5/5 5/5
Finger abduction/adduction	5/5	5/5
Grip strength	Good	Good
Hip-flexor/extension	4/5 5/5	4/5 5/5
Knee-flexion/extension	5/5 5/5	5/5 5/5
Ankle-dorsiflexion/plantar flexion	5/5 5/5	5/5 5/5
Toe extension	5/5	5/5
Sensations	Normal	Normal
Hoffman sign	Positive	Positive
Inverted supinator jerk	Positive	Positive

**Figure 1:**
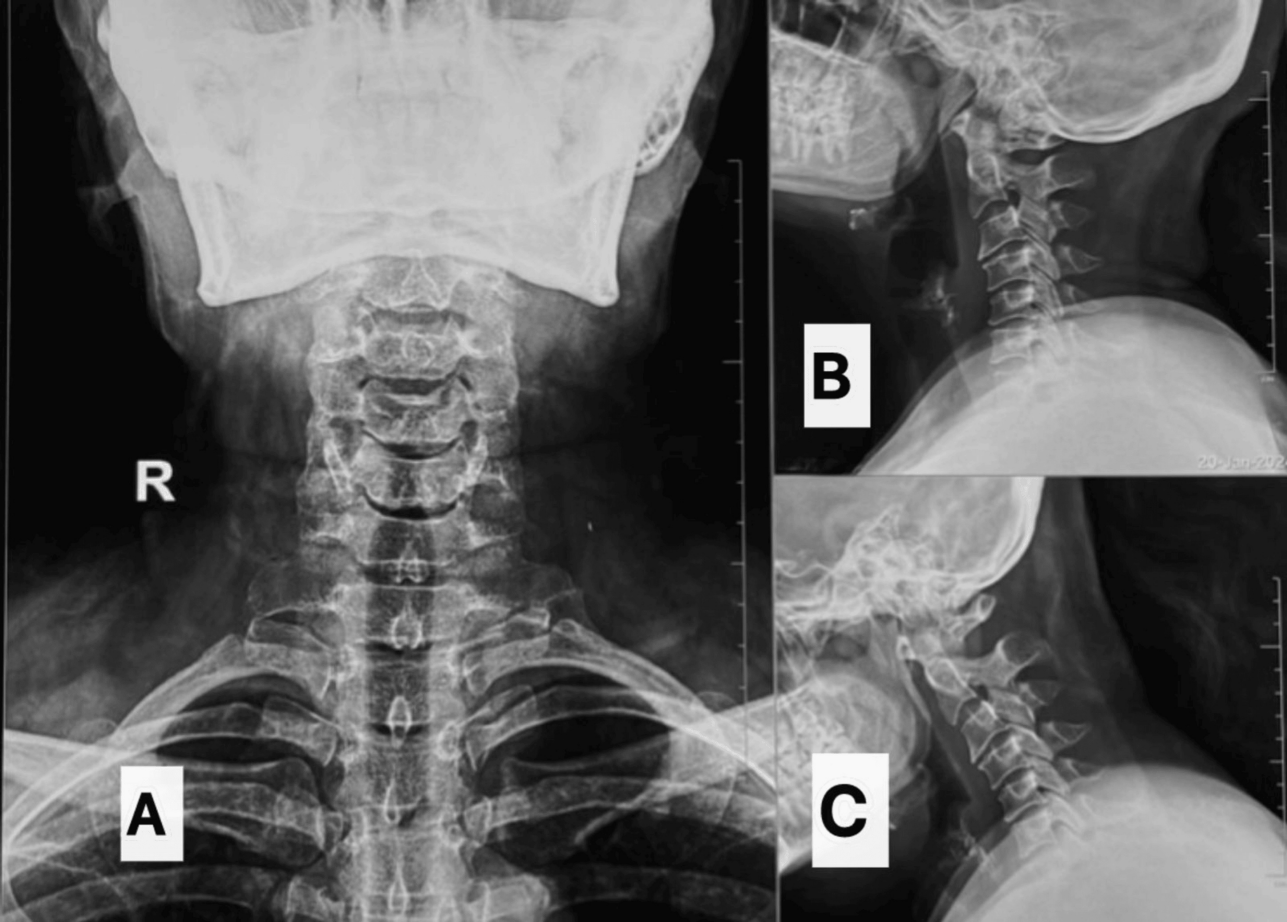
Preoperative cervical anteroposterior (A) and lateral-extension (B)/flexion (C) radiographs showing marginal osteophytes at C3-4 and C4-5 levels with normal disc height and no instability. C3-4: cervical 3-4, C4-5: cervical 4-5

Clinically, we suspected mild cervical myelopathy that had progressed from a Nurick grade 2 toward grade 3 over one month, as the patient showed upper motor neuron (UMN) signs, such as brisk lower limb reflexes and upgoing plantar reflex, along with neck pain and upper limb radiculopathy with loss of dexterity but grossly normal power. Blood investigations revealed a white blood cell (WBC) count of 10,300/mm^3^, a raised C-reactive protein (CRP) of 185 mg/L. Preoperative radiograph showed a normal appearance with no instability (Figures 1A-1C).

**Figure 2:**
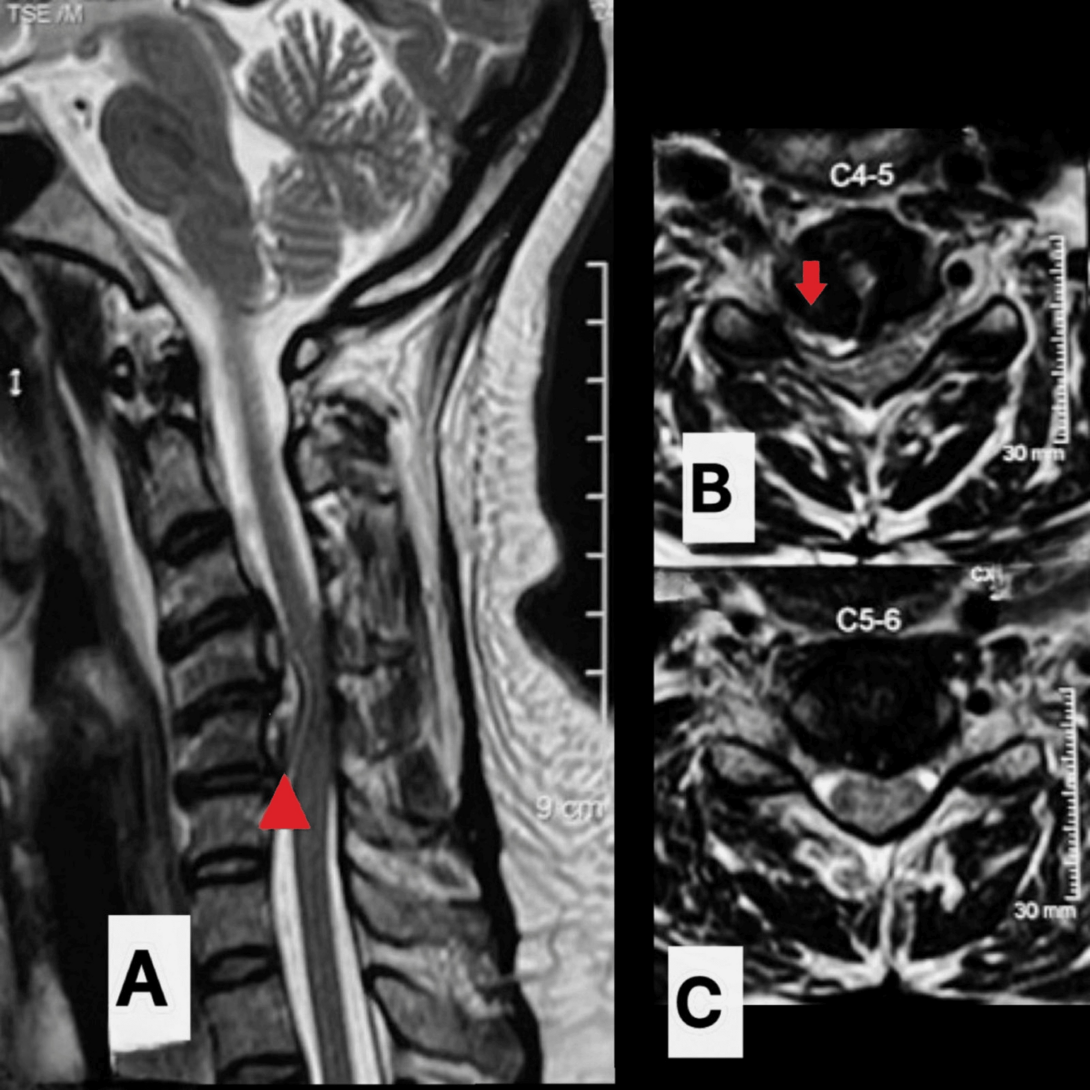
Preoperative T2w cervical MRI showing C4-5 disc signal changes in sagittal section (A) and axial section (B-C) with collection in the anterior epidural space descending behind the C5 vertebral body (marked with a red arrow). MRI: magnetic resonance imaging, T2w: T2-weighted, C4-5: cervical 4-5, C5: cervical 5

Magnetic resonance imaging (MRI) revealed vertebral body edema, signal changes in cervical 4-5 (C4-5) disc with collection in the anterior epidural space descending behind the C5 vertebral body on a T2-weighted (T2w) sagittal film. It was also compressing the cord from C4 to C6 disc space with cord signal changes. MRI report was suggestive of C4-5 spondylodiscitis with anterior epidural abscess with cord edema (Figure 2).

Management

**Figure 3:**
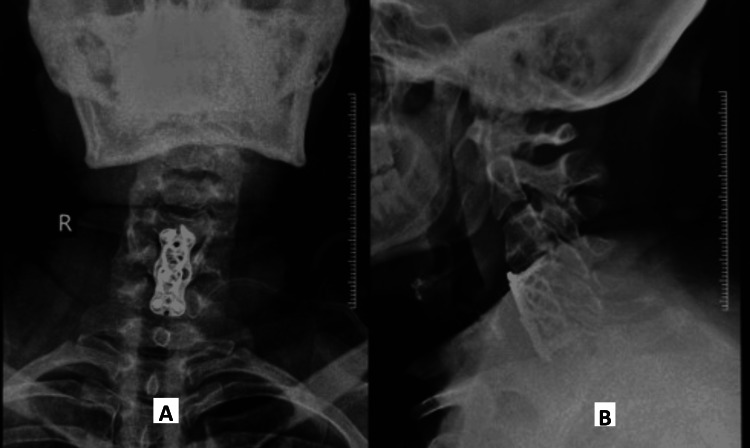
Postoperative day 0 anteroposterior (A) and lateral (B) X-rays showing C5 anterior corpectomy with C4-6 fusion with implant in situ. C4-6: cervical 4-6, C5: cervical 5

We decided to perform a C5 corpectomy with C4-6 fusion using a mesh cage and plate construct to obtain an adequate decompression and stabilization of the segment (Figure 3).

After fluoroscopic confirmation of C4-5 and C5-6 levels, discectomies were performed at both levels. The C5 vertebral body was excised using an ultrasonic bone scalpel (BoneScalpel®; Misonix, Inc., Farmingdale, New York, USA). Intraoperatively, a frank purulent collection was encountered; pus and disc space tissue samples were obtained and sent for histopathological examination and culture sensitivity analysis. A C5 corpectomy was performed, extending from the left to right uncovertebral joints (≈10-12 mm on either side from the midline). Posterior longitudinal ligament (PLL) was excised. The resultant defect was reconstructed with a mesh cage packed with an autologous bone graft. Finally, C4 to C6 vertebral bodies were stabilized with an anterior plate and screws. No cerebrospinal fluid (CSF) leak or evidence of dural adherence was noted intraoperatively. A 12-Fr drain was placed before closure. The procedure was uneventful, and the patient was comfortable postoperatively.

**Figure 4:**
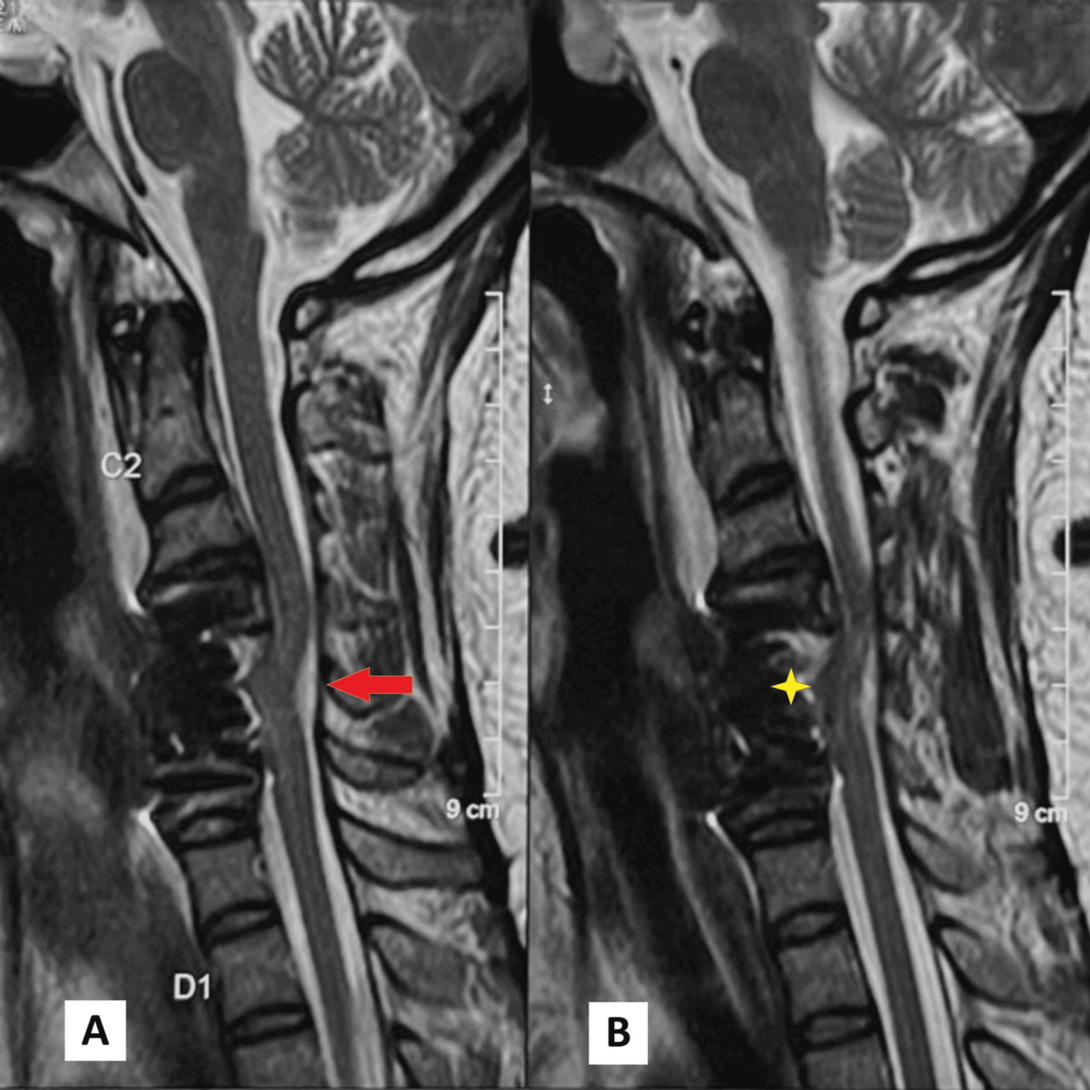
Postoperative day 1 T2w sagittal screening MRI showing ventral cord herniation (VCH) at C5-6 level as suggested by focal anterior angulation of the dura and cord with widened dorsal CSF column (Figure 4A, marked with a red arrow) and tethering of the cord to the anterior aspect of the thecal sac (Figure 4B, marked with a yellow star). MRI: magnetic resonance imaging, T2w: T2-weighted, CSF: cerebrospinal fluid, C5-6: cervical 5-6

The patient was mobilized on day 1 as per the enhanced recovery after surgery (ERAS) protocols [3]. There was no wound soakage or bowel/bladder involvement. The postoperative drain output did not demonstrate any clear fluid suggestive of a CSF leak. On postoperative day 2, the patient developed new-onset mild weakness in the right-hand grip. Neurological examination demonstrated Medical Research Council (MRC) grade 4/5 motor strength in the C7-T1 distribution on the right side with mild numbness along the C7-T1 dermatomal distribution. The remainder of the neurological examination was unchanged. Postoperative sagittal T2w MRI was conducted as a routine protocol to confirm the decompression, which showed ventral displacement of the cord at the C5-6 level as suggested by focal anterior angulation of the cord, tethering of the cord to the anterior aspect of thecal sac, and widened dorsal CSF column (Figures 4A-4B). 

Postoperative day 2 histopathology revealed a tuberculous lesion with acid-fast bacilli, which was confirmed by the detection of rifampicin-sensitive *Mycobacterium tuberculosis* on GeneXpert (GeneXpert® System; Cepheid, Sunnyvale, CA, USA) result. The patient was subsequently started on a 12-month anti-tubercular therapy regimen consisting of an intensive phase with isoniazid (300 mg/day), rifampicin (450-600 mg/day), pyrazinamide (1500-2000 mg/day), and ethambutol (800-1200 mg/day) for two months, followed by a continuation phase with isoniazid, rifampicin, and ethambutol for the remaining 10 months. Drain was subsequently removed on postoperative day 3, and the patient was discharged on anti-Koch's treatment (AKT). In view of the absence of significant neurological deficit or functional limitation, the patient was managed conservatively with planned surveillance.

**Figure 5:**
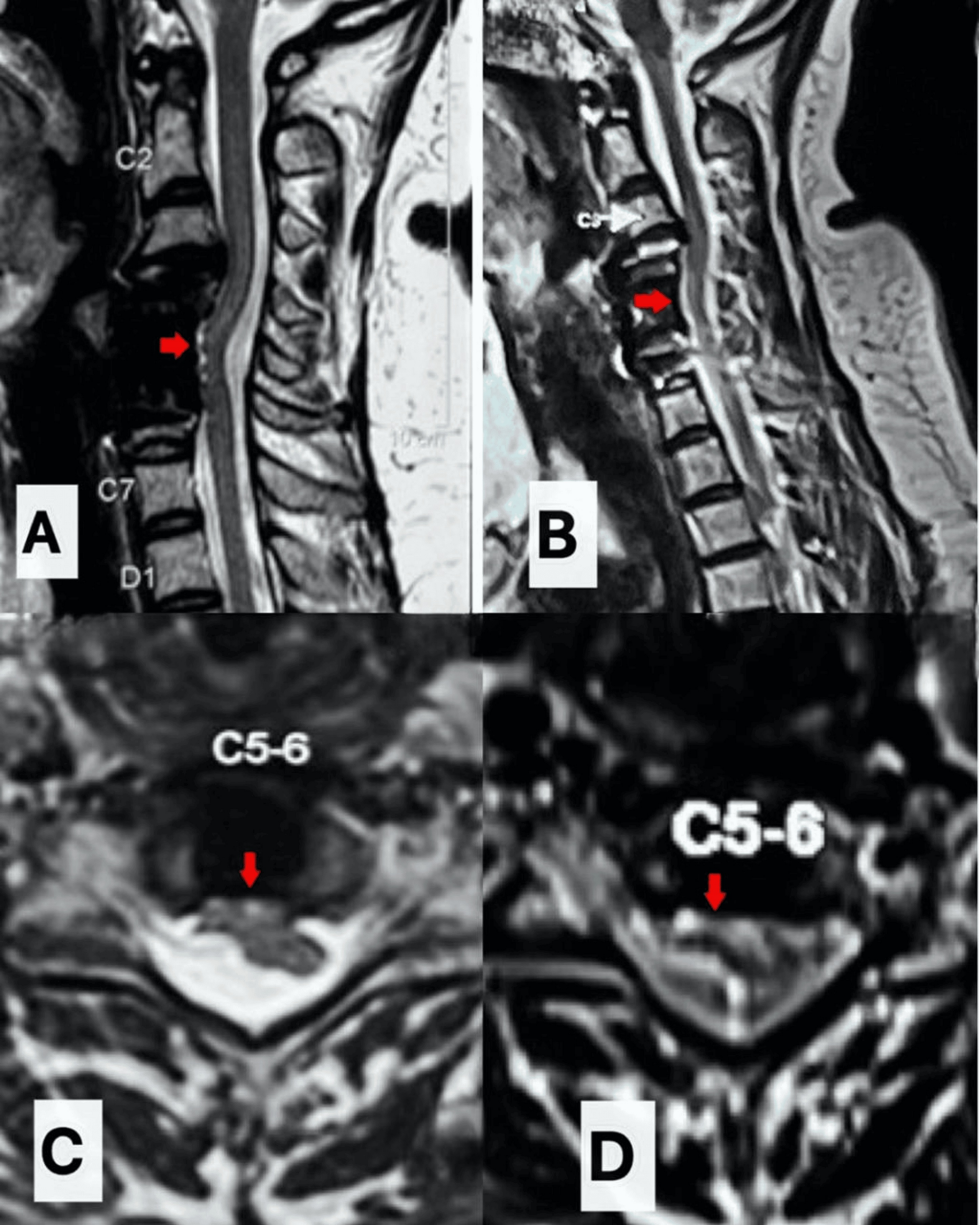
Sequential MRI demonstrating gradual spontaneous restoration of the ventral cerebrospinal fluid (CSF) column. (A) At three months (sagittal), a thin ventral CSF cleft was observed (arrow). (B) At nine months, there was an interval increase in the ventral CSF space, indicating progressive release of anterior tethering. (C, D) Corresponding axial images at three and nine months demonstrate increasing ventral CSF around the cord. These findings suggest gradual spontaneous resolution of ventral cord displacement. MRI: magnetic resonance imaging

The patient was maintained on serial clinical and radiological follow-up to monitor neurological recovery and resolution of infection. Follow-up evaluations were performed at 1, 3, 6, 12, and 24 months after surgery. MRI scans were obtained at three months, nine months, and at the two-year follow-up. The MRI scans at three and nine months demonstrated the appearance of ventral streaks of CSF between the anterior surface of the cord and the corpectomy site (Figures 5A-5D). 

**Figure 6:**
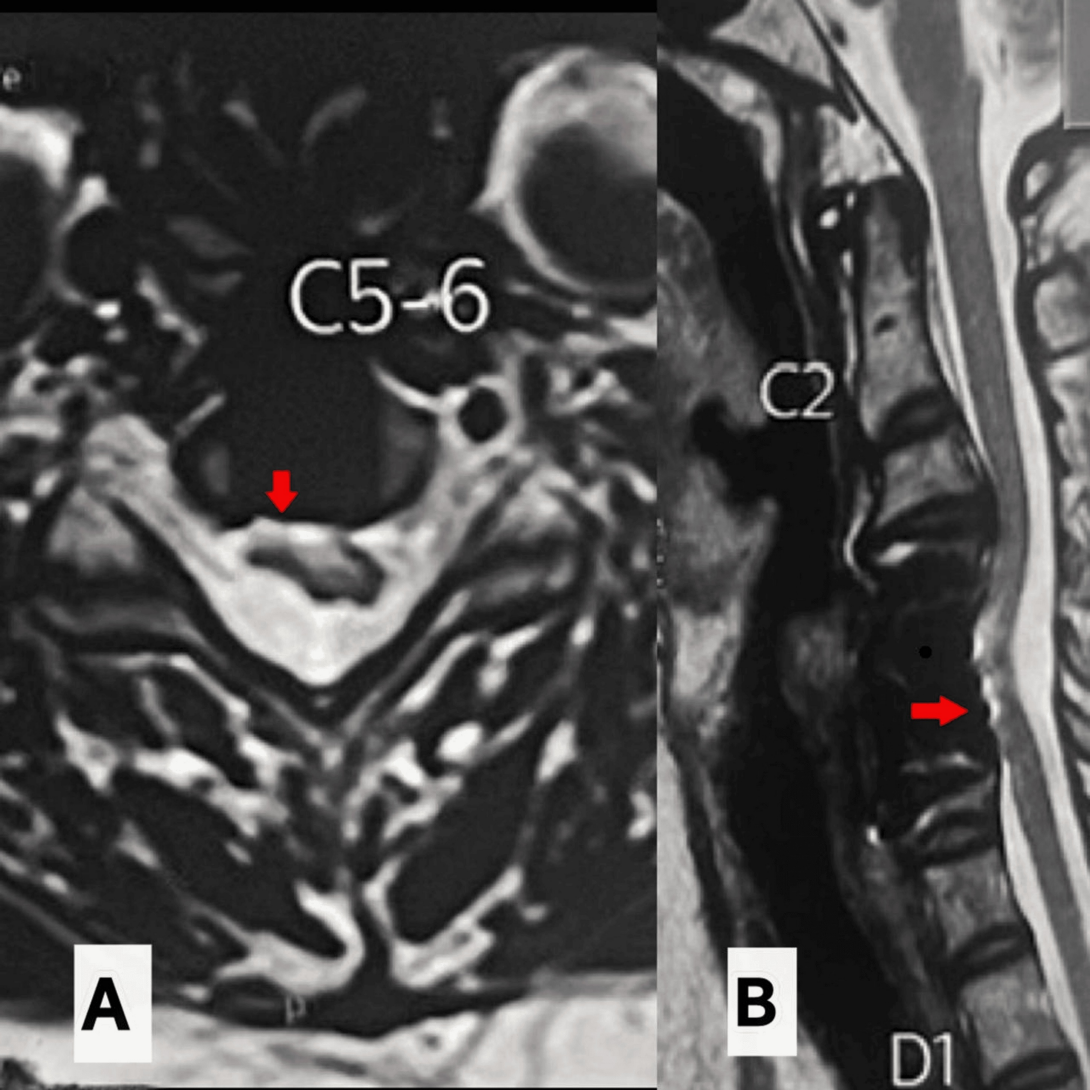
Two-year follow-up MRI demonstrating restoration of the ventral cerebrospinal fluid (CSF) column. (A) Axial image at the C5-6 level shows re-established ventral CSF space with residual cord thinning (arrow). (B) Sagittal image demonstrates restoration of the ventral CSF column at the level of previous ventral displacement, with associated focal myelomalacia and relative widening of the dorsal CSF space (arrow), consistent with chronic remodeling. MRI: magnetic resonance imaging, C5-6: cervical 5-6

At the two-year follow-up, MRI showed restoration of the ventral CSF column along with cord thinning, focal myelomalacia, and widening of the dorsal CSF column at the level of the ventral displacement (Figure 6).

At routine follow-up, the patient continued to experience mild upper-limb pain with numbness, without evidence of progressive myelopathy. The transient grip weakness noted on postoperative day 1 had resolved completely, and no further neurological decline occurred. This clinical improvement was attributed to the timely anterior decompression and fusion of the affected segment, along with initiation of the anti-tubercular regimen. The patient was comfortable doing activities of daily living without any functional limitations.

## Discussion

The documented literature mainly focuses on idiopathic thoracic herniations [4-10]. The number of cases with cervical ventral cord displacement or herniation reported is limited. Cervical involvement is described only in isolated case reports and small series with an incidence of 0.02% [11]. This makes awareness critical, as misdiagnosis may lead to inappropriate anterior or posterior decompressive surgery. Finneran et al. [12] documented the first case of ventral cervical cord herniation in a 17-year postoperative case of staged, corrective anterior C5 corpectomy with posterior cervical fusion from C2-T4 surgery. The patient noted clinical improvement postoperatively over the years. No CSF leak or dural misadventure was noted in the discharge report summary. He presented 17 years later with generalized fatigue for one week. The patient was a known case of gastrointestinal stromal tumor with metastasis, on chemotherapy. He reported a one-year history of neck pain, headache, and paresthesia in the bilateral upper extremities. He was admitted under an oncologist for workup of the same and developed acute-onset quadriparesis. The patient reported multiple such episodes over the past one year, which resolved spontaneously over two-four hours. A head CT scan did not show any acute findings, and cervical spine CT images showed excellent fusion with thinning/resorption of graft at the corpectomy site. An MRI of the cervical spine demonstrated marked anterior spinal cord displacement into the ventral corpectomy site with signal changes within the spinal cord and myelomalacia. The patient underwent an anterior surgical release of the adherent dura to the corpectomy site, combined with plating and bone grafting. Similar to this patient, our case did not demonstrate any dural defect or CSF leak post-surgery. However, unlike this patient, our case did not develop a gross neurological deficit or signs of progressive myelopathy.

Radiological findings, together with the clinical course, excluded potential causes of anterior cord tethering, including epidural fibrosis, graft-dura interface scarring, a demonstrable dural defect, and CSF leakage. Furthermore, the absence of progressive myelopathy in the absence of a dural defect made ventral cord herniation (VCH) unlikely. Accordingly, the condition was interpreted as ventral displacement of the cervical cord. The mild weakness in the right-hand grip resolved over the next two years postoperatively with no new complaints.

Spinal cord herniation is a rare condition with idiopathic, iatrogenic, or post-traumatic etiologies [1]. Idiopathic cases most commonly involve the thoracic spine, typically through an anterior dural defect, and predominantly affect middle-aged females [5,13]. In contrast, post-traumatic and iatrogenic herniations may occur at any level corresponding to the site of injury or surgical intervention. Although the exact pathogenesis remains uncertain, factors such as dural defects, adhesions, CSF dynamics, and spinal alignment are thought to contribute [14]. Most studies show patients presenting with new-onset neurological deficit or progressive myelopathy. Spontaneous resolution is not convincingly documented, thus warranting surgical intervention. Reported mechanisms underlying neurological deficits due to cord herniation include 1) ischemia combined with mechanical compression, 2) tethering of the spinal cord at the site of herniation, and 3) traction-induced distortion and deformation of the cord [15]. Spinal cord herniation preferentially occurs along the concave side of spinal alignment and therefore most often affects the dorsal aspect in the cervical region [16]. Although these mechanisms likely explain the majority of cases, our findings indicate that ventral spinal cord displacement can occur even without a clearly demonstrable dural defect. In this case, no intraoperative or postoperative CSF leak was noted. The pathology was detected incidentally on a postoperative screening MRI, performed as a routine protocol to detect any residual levels of compression and signal changes around the cord. We hypothesize a multifactorial mechanism wherein dural fragility and chronic anterior thecal adhesions to infective tissue create a tethered state. Surgical decompression with corpectomy likely disrupts this equilibrium, producing a relative vacuum effect at the operative site that promotes ventral displacement of the spinal cord. This case highlights the significance of immediate postoperative MRI in patients with myelopathy [17].

Because of biomechanical factors and approach-related considerations, dorsal spinal cord herniations resulting in pseudo-meningocele are reported more often in the literature than ventral herniations [11]; however, only six case reports have shown ventral herniation of the cervical cord after anterior surgery [12,16,18-21]. These studies show multiple anterior surgeries as a risk factor in the development of VCH in patients with myelopathy. In addition to this, multiple studies have shown resection of ossified posterior longitudinal ligament (OPLL) as a precipitating factor in the development of VCH, which can be identified as a double-layer sign characterized by anterior and posterior ossified rims separated by a centrally hypertrophied PLL, as pathognomonic for a diffusely absent dura [19]. Thus, identifying this condition can help improve the patient outcome, and surgeons should be alert in this situation to prevent complications.

Although currently, no data are available on the management of asymptomatic cervical ventral cord displacement, we recommend a close follow-up of the patient to detect any signs of progressive myelopathy.

## Conclusions

Postoperative cervical ventral spinal cord displacement is a rare complication that may occur even without an identifiable dural defect or CSF leak. This case highlights a potential intermediate entity in the spectrum of ventral spinal cord pathology in the setting of spinal tuberculosis and underscores the importance of distinguishing reversible cord displacement from true herniation to avoid unnecessary surgical intervention. In the absence of progressive myelopathy and gross neurological worsening, close clinical and radiological follow-up may be an appropriate management strategy. This case also highlights the role of early postoperative MRI in detecting this entity following anterior cervical surgery.

## References

[1] Aiyer SN, Shetty AP, Kanna R, Maheswaran A, Rajasekaran S (2016). Spinal cord herniation following cervical meningioma excision: a rare clinical entity and review of literature. Eur Spine J.

[2] Berg-Johnsen J, Ilstad E, Kolstad F, Züchner M, Sundseth J (2014). Idiopathic ventral spinal cord herniation: an increasingly recognized cause of thoracic myelopathy. J Cent Nerv Syst Dis.

[3] Bansal T, Sharan AD, Garg B (2022). Enhanced recovery after surgery (ERAS) protocol in spine surgery. J Clin Orthop Trauma.

[4] Vallée B, Mercier P, Menei P (1999). Ventral transdural herniation of the thoracic spinal cord: surgical treatment in four cases and review of literature. Acta Neurochir (Wien).

[5] Darbar A, Krishnamurthy S, Holsapple JW, Hodge CJ Jr (2006). Ventral thoracic spinal cord herniation: frequently misdiagnosed entity. Spine (Phila Pa 1976).

[6] Bakhsheshian J, Strickland BA, Liu JC (2020). Ventral thoracic spinal cord herniation: clinical image and video illustration of microsurgical treatment. World Neurosurg.

[7] Ghali MGZ, Srinivasan VM, Rao VY, Omeis I (2018). Idiopathic thoracic spinal cord herniation. J Clin Neurosci.

[8] Randhawa PS, Roark C, Case D, Seinfeld J (2020). Idiopathic spinal cord herniation associated with a thoracic disc herniation: case report, surgical video, and literature review. Clin Spine Surg.

[9] Bartels RHMA, Kusters B, Brunner H, Hosman AJ, van Alfen N, Grotenhuis JA (2018). Pathogenesis of idiopathic ventral herniation of spinal cord: neuropathologic analysis. World Neurosurg.

[10] Iunes EA, Barletta EA, Suzuki FS (2020). Idiopathic ventral spinal cord herniation: video report and systematic review. World Neurosurg.

[11] Nakashima H, Ishikawa Y, Kato F (2020). Postoperative iatrogenic spinal cord herniation: three case reports with a literature review. Nagoya J Med Sci.

[12] Finneran MM, Schaible K (2020). Ventral herniation of the cervical cord after single-level corpectomy. World Neurosurg.

[13] Ellger T, Schul C, Heindel W, Evers S, Ringelstein EB (2006). Idiopathic spinal cord herniation causing progressive Brown-Séquard syndrome. Clin Neurol Neurosurg.

[14] Espiritu MT, Rhyne A, Darden BV 2nd (2010). Dural tears in spine surgery. J Am Acad Orthop Surg.

[15] Borges LF, Zervas NT, Lehrich JR (1995). Idiopathic spinal cord herniation: a treatable cause of the Brown-Sequard syndrome--case report. Neurosurgery.

[16] Taya S, Chaudhary V, Acharya UV (2024). Postoperative rare presentation of ventral cervical cord herniation: a case report. J Clin Diagn Res.

[17] Dave BR, Anil A, Agrawal S (2025). Prospective analysis of day-one postoperative MRI following cervical decompression for cervical myelopathy: insights into residual compression and signal changes. Cureus.

[18] Sinha S, George KJ (2020). Spinal cord herniation following multilevel anterior cervical discectomy and fusion: a case report and literature review. Surg Neurol Int.

[19] Min JH, Jung BJ, Jang JS, Kim SK, Jung DJ, Lee SH (2009). Spinal cord herniation after multilevel anterior cervical corpectomy and fusion for ossification of the posterior longitudinal ligament of the cervical spine. J Neurosurg Spine.

[20] Kizilay Z, Yilmaz A, Ismailoglu O, Coskun ME (2016). Anterior spinal cord herniation after multilevel anterior cervical corpectomy: a case report. Neurol Neurochir Pol.

[21] Guppy KH, Silverthorn JW (2017). Spinal cord herniation after cervical corpectomy with cerebrospinal fluid leak: case report and review of the literature. World Neurosurg.

